# Regulation of Canonical Oncogenic Signaling Pathways in Cancer via DNA Methylation

**DOI:** 10.3390/cancers12113199

**Published:** 2020-10-30

**Authors:** Jennifer Lu, Premila Wilfred, Darren Korbie, Matt Trau

**Affiliations:** 1 Centre for Personalised Nanomedicine, Australian Institute for Bioengineering and Nanotechnology, The University of Queensland, St Lucia, QLD 4072, Australia; jennifer.lu@uqconnect.edu.au (J.L.); p.wilfred@uq.net.au (P.W.); 2 Australian Institute for Bioengineering and Nanotechnology, The University of Queensland, St Lucia, QLD 4072, Australia; 3 School of Chemistry and Molecular Biosciences, The University of Queensland, St Lucia, QLD 4072, Australia

**Keywords:** DNA methylation, machine learning, cancer biomarkers, gene expression, p53, NRF2, Wnt, Hippo, signaling pathways

## Abstract

**Simple Summary:**

Aberrant epigenetic modifications in oncogenic pathways can lead to the onset of different cancers. This study aims to explore the role of differential DNA methylation in the regulation of oncogenic signaling pathways by integrating data from multiple sources including methylome, transcriptome and clinical presentation to uncover the effect of methylation changes acting on the four most common cancers. We utilized a differential methylation-parsing pipeline, which extracted differentially methylated biomarkers based on feature selection. Extracted biomarkers were integrated with the matching RNA-Seq and clinical data to determine if these differentially methylated CpGs could serve as potential diagnostic candidates for the four most common cancers. Our results suggested differential methylation of the genes within the NRF2-PI3K pathway may lead to the presentation of various cancer and serve as potential epigenetic biomodifiers.

**Abstract:**

Disruption of signaling pathways that plays a role in the normal development and cellular homeostasis may lead to the dysregulation of cellular signaling and bring about the onset of different diseases, including cancer. In addition to genetic aberrations, DNA methylation also acts as an epigenetic modifier to drive the onset and progression of cancer by mediating the reversible transcription of related genes. Although the role of DNA methylation as an alternative driver of carcinogenesis has been well-established, the global effects of DNA methylation on oncogenic signaling pathways and the presentation of cancer is only emerging. In this article, we introduced a differential methylation parsing pipeline (MethylMine) which mined for epigenetic biomarkers based on feature selection. This pipeline was used to mine for biomarkers, which presented a substantial difference in methylation between the tumor and the matching normal tissue samples. Combined with the Data Integration Analysis for Biomarker discovery (DIABLO) framework for machine learning and multi-omic analysis, we revisited the TCGA DNA methylation and RNA-Seq datasets for breast, colorectal, lung, and prostate cancer, and identified differentially methylated genes within the NRF2-KEAP1/PI3K oncogenic pathway, which regulates the expression of cytoprotective genes, that serve as potential therapeutic targets to treat different cancers.

## 1. Introduction

Over the past few decades, the field of cancer research has continuously evolved [1]. Different techniques have been applied in the detection and treatment of cancer, including screening for cancers during early stages of disease, in order to identify different cancers before the onset of symptoms [2,3,4]. Furthermore, new strategies to predict the outcome of cancer treatments has also emerged [5,6,7]. With the advent of new technologies in the field of oncology, large amounts of cancer data have collected (including genomic, transcriptomic and epigenomic data). However, the accurate prediction of disease outcome and differentiation between different cancers remains one of the most interesting and challenging tasks for researchers. Machine-learning (ML) methods have become a popular tool in recent years to assist medical researchers to discover and identify patterns and relationships between different cancers, in order to create more effective biomarker panels which will be able to predict potential biomarkers that are present in different cancers [8,9,10,11,12]. ML defines the ability of machines to learn and predict future events and outcomes based on large datasets, and has been utilized in health care to improve the interpretation of medical data especially in the development of novel computational tools for stratification, grading, and prognostication of patients with the goal of improving patient care [8,13,14].

Genomic data has been associated with rich clinical annotations, and DNA sequencing has now been incorporated into standard clinical practice [15]. An emerging trend in application of machine learning in oncology is the integration and analysis of mixed data (e.g., genomic, transcriptomic, and epigenomics)—multi-omics to uncover patterns that are reflected across the different data. These studies have painted a portrait of the mutation landscape of multiple cancers including breast, colorectal, lung and prostate [16,17,18,19]. However, some cancers may have relatively few genetic mutations, with their biology largely driven by other types of variations such as aberrant epigenetic modifications.

Epigenetics is the study of heritable phenotypic changes that do not involve alteration in the genomic sequence, and includes mechanisms such as histone modification, altered micro (mi) RNA, or long non-coding (lnc) RNA [20]. Mediated by DNA methyltransferases, DNA methylation is a major epigenetic mechanism that occurs when a methyl group is transferred to the C5 position of the carbon to form 5-methylcytosine (5mC). This modification plays an essential role in various biological processes including the regulation of gene expression, genomic imprinting, cell differentiation and normal development [21,22,23]. Aberrant DNA methylation has been associated with various diseases, including cancers [24,25,26]. DNA hypermethylation acts as a regulator of gene expression, and methylation-mediated silencing of tumor suppressor genes or regulatory regions within the genome can lead to dysregulation of cell growth or altered response to cancer therapies [27,28,29,30]. Despite the varied and complex nature of modifications in the epigenetic landscape, many cancers display a high degree of similarity both across different tissues and within the tissues of origin [31,32]. Therefore, abnormal DNA methylation has been viewed as an attractive avenue for development of cancer diagnostics and therapeutics.

Although thousands of studies have highlighted the value of using changes in DNA methylation as candidate biomarkers in the detection and treatment of various cancers, only a handful of these targets have been approved for clinical use (as summarized in Table 1). Traditionally, these studies have focused on the association of differential methylation and its effects on individual genes in the phenotypic presentation of different carcinogenesis. However, few studies have investigated the effects of epigenetic changes (especially DNA methylation) on individual and complex signaling pathways across cancers at different anatomical sites. Cell signaling (signal transduction) plays a vital role within biological systems by relaying extracellular signals in order to regulate intracellular gene expression. The signal transduction process is typically initiated when a ligand binds to a membrane-bound receptor, triggering a cascade of intercellular signaling activities through activation of multiple kinases. This ultimately has an effect on the downstream gene expression activation of different signaling pathways and can lead to various physiological or cellular responses (e.g., cell proliferation, differentiation, and metabolism), and any disruptions within these intra and/or extracellular communication chains can lead to the development of different diseases, including cancer [33,34,35]. As DNA methylation is a dynamic and reversible process, changes in methylation could act as regulators of certain oncogenic pathways, leading to development of diseases, and provide an attractive target for development of biomarkers and therapies.

Pathways and network analysis refer to any analytical techniques that benefit from biological or molecular network information to gain insight into a biological system. The main goal through these studies is to reduce data involving thousands of altered genes and proteins into a smaller and more interpretable set of altered process by grouping genes with similar processes together [52]. This process-oriented analysis can generate testable hypothesis, identify druggable targets, and tumor subtypes with clinically distinct outcomes, and identify mechanisms that are either cancer-specific or shared across multiple cancers. Both pathway and network analysis consist of interacting genes, proteins, and other biomolecules that carry out biological functions. Pathways are small-scale systems of well-studied processes where the interactions consist of consensus biochemical reactions and events of regulation and signaling based on decades of research that can be visualized via a directed acyclic graph (DAG) [53]. In contrast, network interactions analysis contains simplified abstractions of complex cellular organizations. Although network enrichment plots are noisy and challenging to visualize and interpret, they contain novel information not covered in well-defined pathways by themselves [54]. Additionally, a related concept to pathway and network analysis is the examination of functionally annotated gene sets that comprise all genes involved in a particular process or pathway without the actual interactions between different genes [55].

Given the rising significance of personalized medicine and the growing trend on the application of ML techniques in cancer research, in this article, we aimed to identify DNA methylation biomarkers that could act as epigenetic modifiers of oncogenic signaling pathways in the presentation of four of the most common cancers—breast, colorectal, lung and prostate [56]. The Cancer Genome Atlas (TCGA) has been extensively mined in the past for pan-cancer studies, as the initiative has curated over 20,000 primary cancer and matched normal samples spanning 33 cancer types [57]. However, many of the previous studies have focused on gene expression data [58,59,60], as opposed to DNA methylation; with even fewer studies taking a pan-cancer approach to exploring the effects of DNA methylation on disrupted oncogenic signaling pathways. Given this, we sought to revisit the TCGA dataset with a view of incorporating both RNA-Seq and DNA methylation data together as an integrated, multi-omic analysis.

In this study, we probed into the methylation of four canonical oncogenic signaling pathways using the same TCGA dataset in order to identify aberrant methylation patterns that may be involved in the disruption of the associated pathways leading to oncogenesis in the four most common cancers (i.e., breast, colorectal, lung, and prostate). Furthermore, a pipeline that identified epigenetic biomarkers with distinct methylation profiles (between specific cancers or tissues) through a supervised feature selection method was used to screen for differentially methylated biomarkers that are involved in the disruption of different pathways. Written in the Python language, the MethylMine pipeline classified biomarkers that are either hypo- or hypermethylated based on the 1st and 3rd quartiles, which are computed from the combined DNA methylation profile of all patient samples corresponding to a specific cancer or tissue type (i.e., tumor or normal tissue).

Extracted biomarkers were integrated with the matching RNA-Seq and clinical data, and then examined using a ML-based application to reflect on potential signaling pathways that might emerge as potential ‘epigenetic switches’ which could then be used as potential therapeutic targets for diagnosing and/or treating tumorigenesis. Using this methodology, we identified a number of CpG sites which are differentially methylated and discuss the potential of using these biomarkers as screening tools for disruptions in the NRF2-PI3K oncogenic signaling pathway, which acts as the major regulator of cytoprotective responses to both endogenous and exogenous stresses from reactive oxygen species (ROS), and serve as potential epigenetic biomodifiers in the diagnosis and treatment of the screened cancers.

## 2. Materials and Methods

### 2.1. Dataset and Preprocessing

Datasets of the four most common cancers [61] were downloaded from The Cancer Genome Atlas from the NCI Genomic Data Commons portal (https://portal.gdc.cancer.gov/, accessed 2018). We downloaded two types of publicly-available data—methylation (beta value of methyl 450k array), and gene expression data (fpkm) using the gdc-client tool [62] (summary of total samples and variables summarized in Table 2). The beta (β) value represents a quantitative measure of the DNA methylation level of specific CpG sites for each sample and ranges from 0 (completely unmethylated) to 1 (completely methylated), while the fpkm represents the number of fragments per kilobase of exon model per million reads that is mapped to the reference genome. For this study, matrices of each cancer depending on the state of the sample (i.e., primary tumor and [solid] normal tissue) were extracted and merged using a custom “matrix merge” script written in Python 3.8 (available on github—https://github.com/uqjlu8/MethylMine).

### 2.2. Overview of the MethylMine Framework

**Figure 1:**
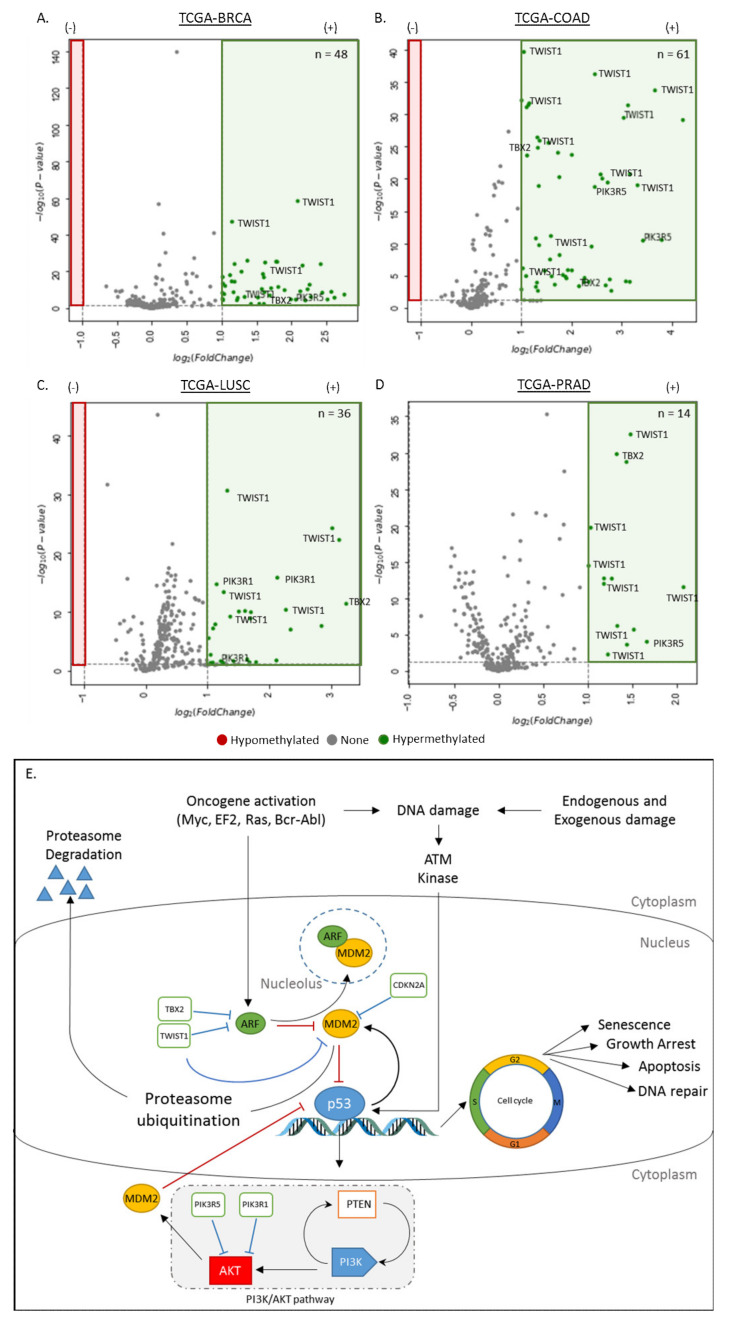
Hypermethylated genes in the dysregulation of the ARF-MDM2-p53 pathway in cancer. (**A**–**D**) Volcano plots showing the hypermethylation of breast; n_tumor_ = 785, n_normal_ = 95), colorectal (n_tumor_ = 313, n_normal_ = 38), lung (n_tumor_ =370, n_normal_ = 42) and prostate cancers (n_tumor_ = 502, n_normal_ = 50) in the ARF-MDM2-p53 pathway. The x-axis is log_2_ fold change of DNA methylation (beta values), whereas the y axis shows the −log_10_ of the *p* values for each CpG site representing the strength of the association. Each point represents a CpG site (CpG = 310). Dashed lines indicated cutoffs for significance, where *p* < 0.05 and log_2_ (Fold Change) > 1, where the number of hypermethylated CpGs in each cancer type is shown in green. Using the current parameters, no hypomethylated CpG sites were observed in the ARF-MDM2-p53 pathway. (**E**) Outline of the ARF-MDM2-p53 pathway, where the major interactions are outlined. During homeostasis, p53 is regulated by MDM2, which mediates the attachment of ubiquitin molecules and transports it to the cytoplasm for proteasome degradation via the proteasome ubiquitination pathway. Deregulation of p53 MDM2 is crucial for an activated p53 response during stress situations. Exposures to endogenous and exogenous damage (e.g., radiation, ultraviolet, genotoxic drugs, etc.) and many DNA-damaging stressors activate several kinases, including ATM kinase, leading to the modification of p53 and inhibiting their interactions with one another, resulting in the destabilization of p53. Overexpression and activation of oncogenes (e.g., Myc, EF2, Ras, Bcr-Abl) stimulate the production of ARF, which sequesters MDM2 into the nucleolus, thus preventing the degradation of p53. Hypermethylation of TBX2, TWIST1, CDKN2A may disrupt this process by repressing the expression of ARF2 and MDM2, respectively. Finally, hypermethylation of PIK3R1 and PIK3R5 may regulate the expression of the AKT gene, which also interacts with the MDM2 gene in the above pathway.

The MethylMine pipeline was written in the Python (3.8+) language, with the aid of other scientific libraries including: matplotlib [63], numpy [64], and pandas [65]. An overview of the MethylMine framework is illustrated in Figure 1. MethylMine was used to filter for CpGs that were differentially methylated between the four cancers by doing quartile comparisons between the different cancer samples. Corresponding RNA-Seq transcripts (fpkm) were extracted using a custom Python script for data integration and further analysis (script available in github—https://github.com/uqjlu8/MethylMine).

### 2.3. Integrated Data Analysis

Transcriptome and methylation data integration (including PLS-DA and correlation studies) were conducted using the Data Integration Analysis for Biomarker discovery (DIABLO) feature within the Bioconductor mixOmics [66] package (version 6.12.1) in the R 4.0.2 (https://www.R-progject.org). The tuning of the component and variable number was performed according to the tutorial at http://mixomics.org. The R script used to perform this analysis is available via github (https://github.com/uqjlu8/MethylMine).

### 2.4. Statistical Analysis

Semi-unsupervised clustering: the methylation values of each sample from the TCGA were obtained as beta values, which represent the level of methylation at a single CpG site (0—no methylation, 1—complete methylation). Semi-unsupervised hierarchical clustering was performed on the probe sites across the four cancer types (both tumor and normal samples) that were found to be differentially methylated by the MethylMine pipeline using the “Euclidean” method via a custom Python (3.8) script using the numpy and seaborn modules.

Interaction network: protein-protein interaction network of proteins correspond to CpG sites that were found to be differentially methylated between the four cancer types—breast, colon, lung and prostate were constructed using the online STRING program [67] using the instructions provided at https://string-db.org/.

Methylation comparison: differentially methylated CpG-sites (β) between normal tissue and the primary tumor of four cancer types were identified using a custom Python script, which firstly calculates the significance between the primary tumor and normal tissue of each cancer type using the non-parametric Mann–Whitney’s test. The log10(*p* value) and −log(fold change) between the tumor and normal tissue of each cancer type was visualized on a volcano plot to examine the difference in methylation of individual CpG sites using the volcano function within the Python bioinfokit module within Python where the cutoffs are: *p* < 0.05 and the −log_2_ (fold change) > 1 (hypermethylation) or <−1 (hypomethylation).

### 2.5. Enrichment Analysis

Enrichment analysis was performed for transcriptomic targets using the online tool g: Profiler (g:GOSt)—https://biit.cs.ut.ee/gprofiler/gost [68], with the following options: Organism: *Homo sapiens*; Statistical domain scope: only annotated genes; Significance threshold: Benjamini–Hochberg FDR; User threshold: 0.05; Numeric IDs treated as: ENTREZGENE_ACC. The following data sources were selected—Gene Ontology: GO molecular functions, GO biological process, Biological pathways: KEGG, Reactome. A generic enrichment map (GEM) was created, and visualized in the Cytoscape software [69] using the EnrichmentMap plugin.

### 2.6. Code Availability and Implementation

The MethylMine pipeline was implemented in the Python language and is available to the public through our github page—https://github.com/uqjlu8/MethylMine. Instructions on implementation of pipeline is available through the github page. Input matrix files (beta) need to be presented in the following format. Although we only presented the filtering of four different cancer types in this manuscript, the current version of the MethylMine pipeline can filter up to 12 cancer types simultaneously.

The DIABLO framework was implemented in the mixOmics R Bioconductor package and is downloadable through their website—http://mixomics.org. Instructions on implementation of DIABLO is available through the mixomics webpage or through CRAN—https://cran.r-project.org/.

The Cytoscape software is available for download through the Cytoscape website—https://cytoscape.org/. Tutorials for implementation of Cytoscape is available through their webpage.

### 2.7. Data Availability

The TCGA datasets analyzed in this article is publically available through the GDC Data Portal—https://portal.gdc.cancer.gov/.

## 3. Results

### 3.1. Differentailly Methylated Genes in the Disruption of Canonical Signalling Pathways

An understanding of the genes and pathways altered in different cancers is essential to identify potential therapeutic options for diagnosing and treatment of tumorigenesis. In this article, we identified several oncogenic signaling pathways that have been recognized to be genetically altered in most cancers. In this section, we investigated the methylation profile of genes associated with four oncogenic signaling pathways (ARF-MDM2-p53, NRF2-KEAP1 and PIK3, Hippo and Wnt, and retinoblastoma (RB)) that were found to be disrupted in most cancers, and identified methylated genes which could serve as potential targets for cancer therapeutics.

#### 3.1.1. Differentially Methylated Genes in the Dysregulation of the ARF-MDM2-p53 Pathway

To identify differential methylation in ARF-MDM2-p53 pathway-associated genes, DNA methylation data (i.e., β values) from primary tumor and corresponding normal tissue samples were extracted for comparative analysis. This analysis focused on the CpGs within the promoter regions (i.e., CpG islands) of the four analyzed cancers (Figure 1), and identified the following number of hypermethylated locis (FDR < 0.05, fold change > 1): TCGA-BRCA (n = 38), TCGA-COAD (n = 61), TCGA-LUSC (n = 36), and TCGA-PRAD (n = 14) that were annotated to six genes. PIK3R5, TBX2 and TWIST1 gene were found to be hypermethylated in all four cancers, while CDKN2A (ARF) is hypermethylated in colorectal and prostate cancers. Additionally, PIK3R1 and PIK3R2 were found to be hypermethylated, specifically in lung and colorectal cancers, respectively.

A survey of literature suggests the hypermethylation of the associated proteins, including PIK3R5, TBX2 and TWIST1 individually, have multiple biological functions in various cancers [70,71], and could serve as potential methylation targets to screen for onset of different cancers. Additionally, the detection of hypermethylated PIK3R5, TBX2 and TWIST1 in all four tumor tissues suggests the specificity of these biomarkers for the four TCGA cancers screened in this manuscript, and their potential as therapeutic targets.

#### 3.1.2. Differentially Methylated Genes in the Disruption of the NRF2-KEAP1 and PIK3K Pathway

**Figure 2:**
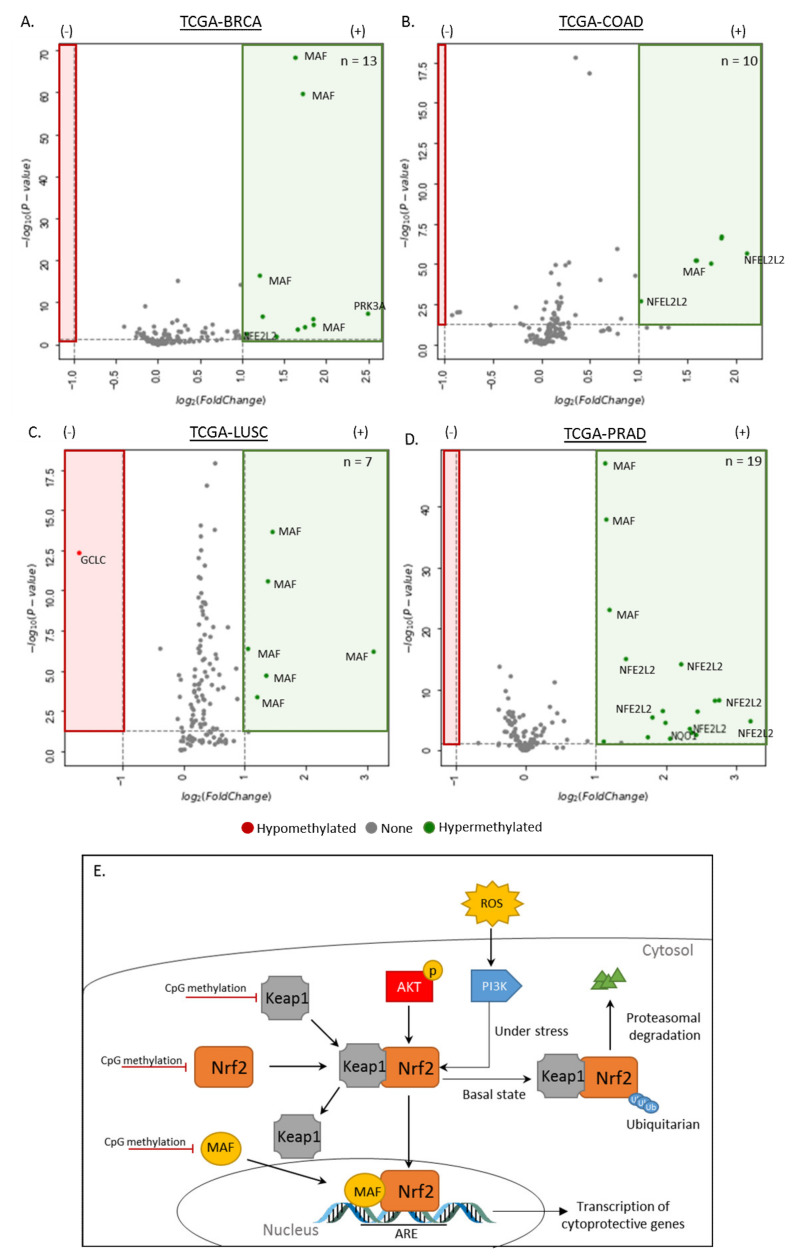
Hypermethylated genes in the dysregulation of the NRF2-KEAP1 and PI3K pathway in cancer. (**A**–**D**) Volcano plots showing the hypermethylation of breast (TCGA-BRCA; n_tumor_ = 785, n_normal_ = 95), colorectal (n_tumor_ = 313, n_normal_ = 38), lung (n_tumor_ =370, n_normal_ = 42) and prostate cancers (n_tumor_ = 502, n_normal_ = 50) in the NRF2-KEAP1 and PI3K pathway. The x-axis is log_2_ fold change of DNA methylation (beta values), whereas the y-axis shows the −log_10_ of the *p* values for each CpG site representing the strength of the association. Each point represents a CpG site (CpG = 52). Dashed lines indicated cutoffs for significance, where *p* < 0.05 and log_2_(Fold Change) > 1 (hypermethylation) or log_2_ (Fold Change) < −1 (hypomethylation). The number of hypermethylated CpGs in each cancer type is shown in green, and hypomethylated CpG sites are shown in red. (**E**) Schematic of NRF2-KEAP1 and PI3K signaling pathway. NRF2 is regulated by KEAP1. During basal (homeostasis) state, NRF2 is continuously ubiquitinated through KEAP1 and undergoes proteasomal degradation. Under stress conditions, KEAP1 is released and the free NRF2 is translocated into the nucleus, where NRF2 binds to the antioxidant response element (ARE) sites within regulatory sites of antioxidant and detoxification genes, including KEAP1 and NRF2. Hypermethylation of promoter-associated CpG sites in either the KEAP1 or NRF2 genes have been shown to play a regulatory role in the regulation of the KEAP1-NRF2 complex and increase the cancerous activity of the NRF2 activity as a result of the reduction in expression of associated mRNA. Additionally, hypermethylation of the MAF gene also interferes with the activity of the NRF2 protein.

To identify differential methylation in the NRF2-KEAP1 and PIK3K pathway associated genes, DNA methylation data (i.e., β values) from primary tumor and corresponding normal tissue samples were extracted for comparative analysis. This analysis focused on the CpGs within the promoter regions (i.e., CpG islands) of the four analyzed cancers (Figure 2), and identified the following number of hypermethylated locis (FDR < 0.05, fold change > 1): TCGA-BRCA (n = 13), TCGA-COAD (n = 10), TCGA-LUSC (n = 7), and TCGA-PRAD (n = 19) that were annotated to three genes. A single hypomethylated site (FDR < 0.05, fold change < −1) was identified in the lung cancer. The NFEF2L2 (NRF2), MAF genes were found to be hypermethylated in all four cancers, while the hypermethylation of NQO gene was only observed in prostate cancer. Additionally, the GCLC gene, which was found to be hypomethylated in lung cancer samples, has been associated with tumor progression and drug resistance. Given only one GCLC-associated CpG site was hypomethylated, the effects on the actual gene may be minimal and require further investigation.

#### 3.1.3. Differentially Methylated Genes in the Disruption of the Hippo and Wnt Pathway

**Figure 3:**
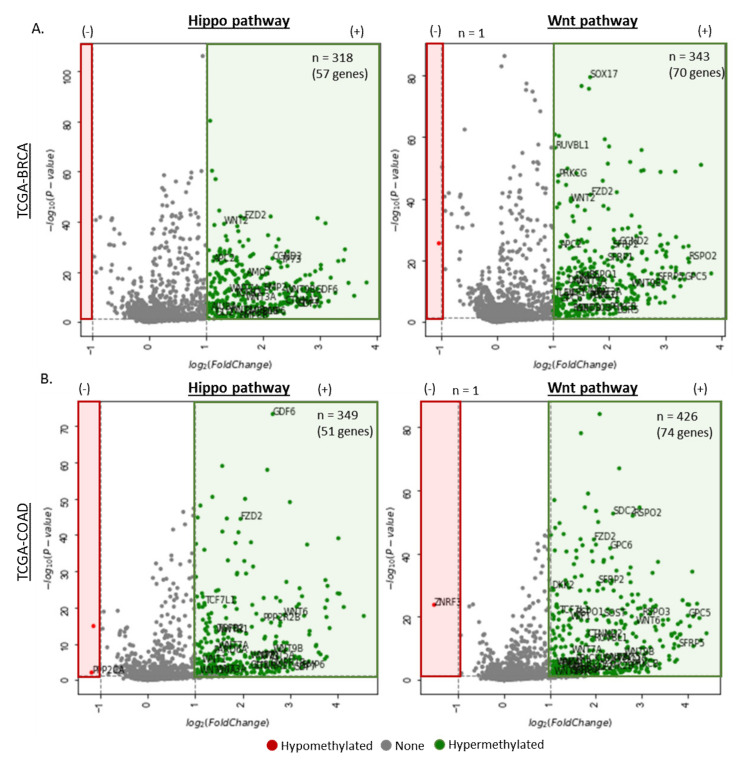

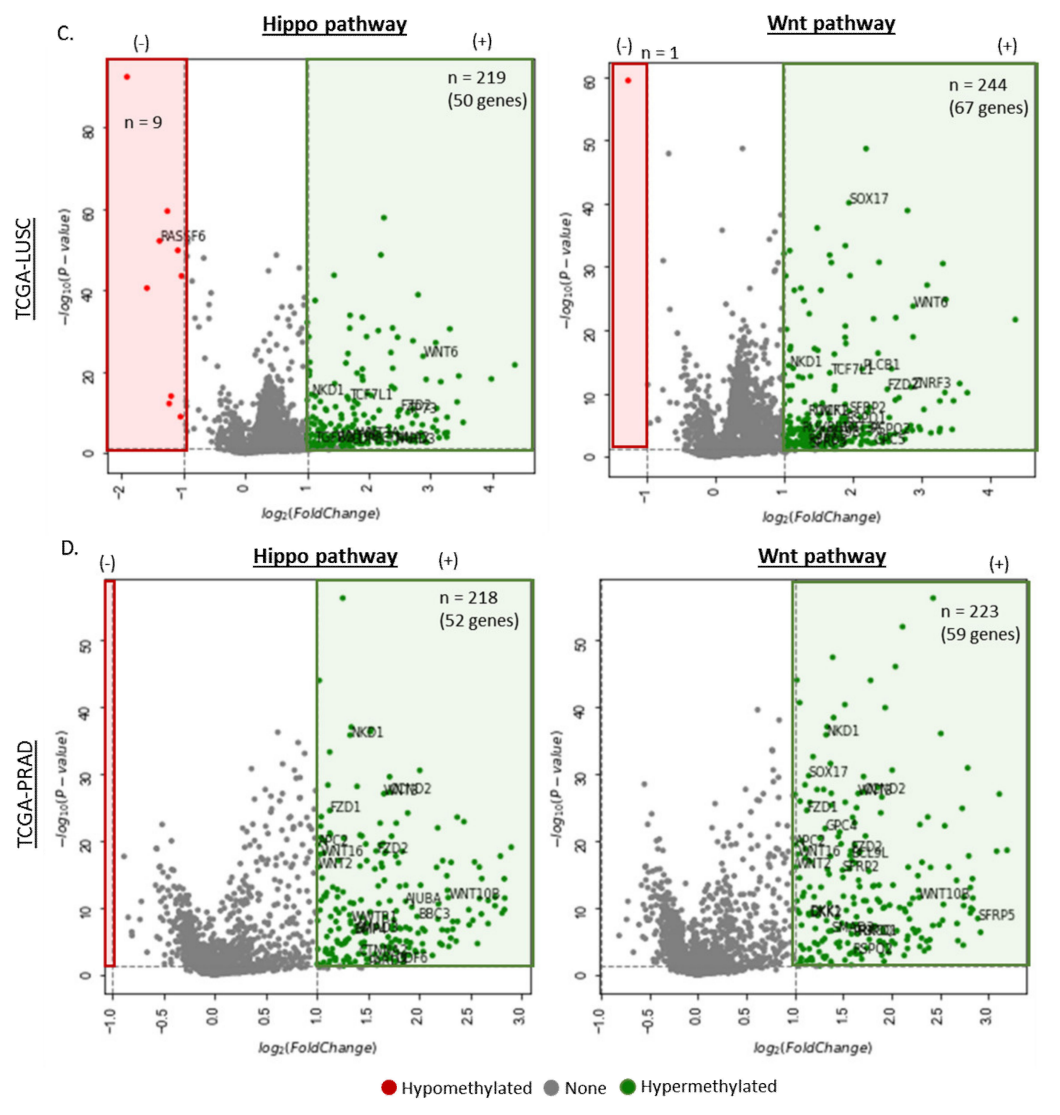
Volcano plots for differential DNA methylation in four different cancers in the Hippo and Wnt pathways. The x-axis is log_2_ fold change of DNA methylation (beta values), whereas the y-axis shows the –log_10_ of the *p* values for each CpG site representing the strength of the association. Each point represents a CpG site. Dashed lines indicated cutoffs for significance, where *p* < 0.05 and log_2_ (Fold Change) < −1 (hypomethylation) or > 1 (hypermethylation). Changes in methylation of CpG island-associated CpG sites are shown for Hippo (left) and Wnt (right) pathway-related CpG sites where (**A**) TCGA-BRCA (n_tumor_ = 785, n_normal_ = 95), (**B**) TCGA-COAD (n_tumor_ = 313, n_normal_ = 38), (**C**) TCGA-LUSC (n_tumor_ = 370, n_normal_ = 42), and (**D**) TCGA-PRAD (n_tumor_ = 502, n_normal_ = 50), where the number of hypermethylated CpGs in each cancer type is shown in green, and the number of hypomethylated CpGs in each cancer type is shown in red. The number of hypermethylated CpGs and number of corresponding genes is also shown.

The Hippo and Wnt signaling pathways play a crucial role in maintaining tissue homeostasis and organ size by orchestrating cell proliferation, differentiation, and apoptosis. These pathways are highly complex and are frequently dysregulated in human cancers. To identify differential methylation in the Hippo and Wnt pathway associated genes, DNA methylation data (i.e., β values) from primary tumor and corresponding normal tissue samples were extracted for comparative analysis. This analysis focused on the CpGs within the promoter regions (i.e., CpG islands) of the four analyzed cancers (Figure 3), and identified the following number of hypermethylated locis (FDR < 0.05, fold change > 1): TCGA-BRCA (n_hippo_ = 318, n_wnt_ = 343), TCGA-COAD (n_hippo_ = 349, n_wnt_ = 426), TCGA-LUSC (n_hippo_ = 219, n_wnt_ = 244), and TCGA-PRAD (n_hippo_ = 218, n_wnt_ = 223).

Genes that are commonly hypermethylated across the four cancers in the Hippo and Wnt pathways include APC, CCND2 FZD, TCFs and WNTS (2, 3, 3A, 5A, 7A, and 9B), suggesting a link between the two pathways during oncogenesis. Furthermore, the hypomethylation of RASSF6 protein in lung cancer samples was observed (Figure 3C), which was in contrast to current literature, which suggests the hypermethylation of RASSF6 genes leads to the poor prognosis in colorectal cancer.

#### 3.1.4. Differentially Methylated Genes in the Disruption of the RB Tumor Suppressor Pathway

**Figure 4:**
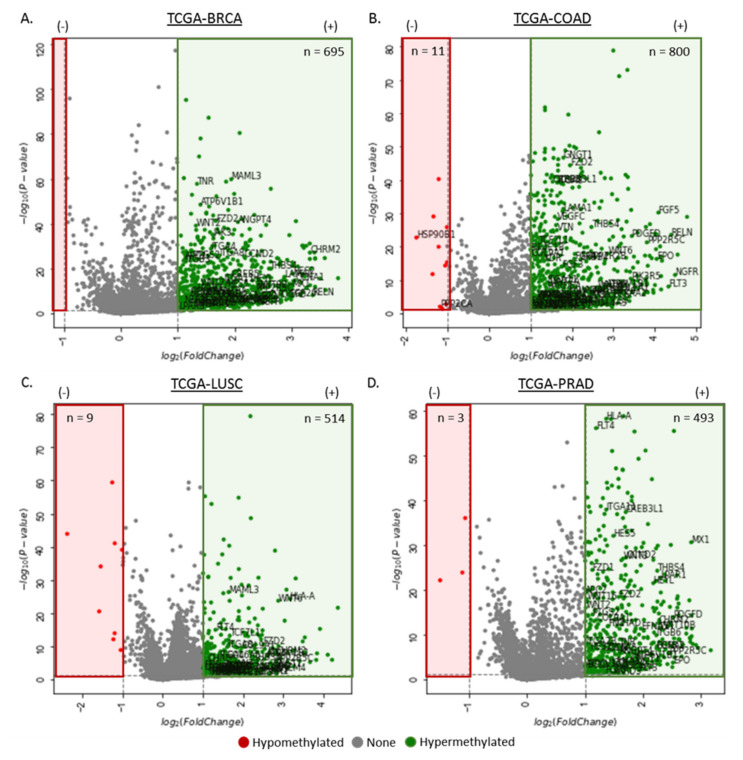

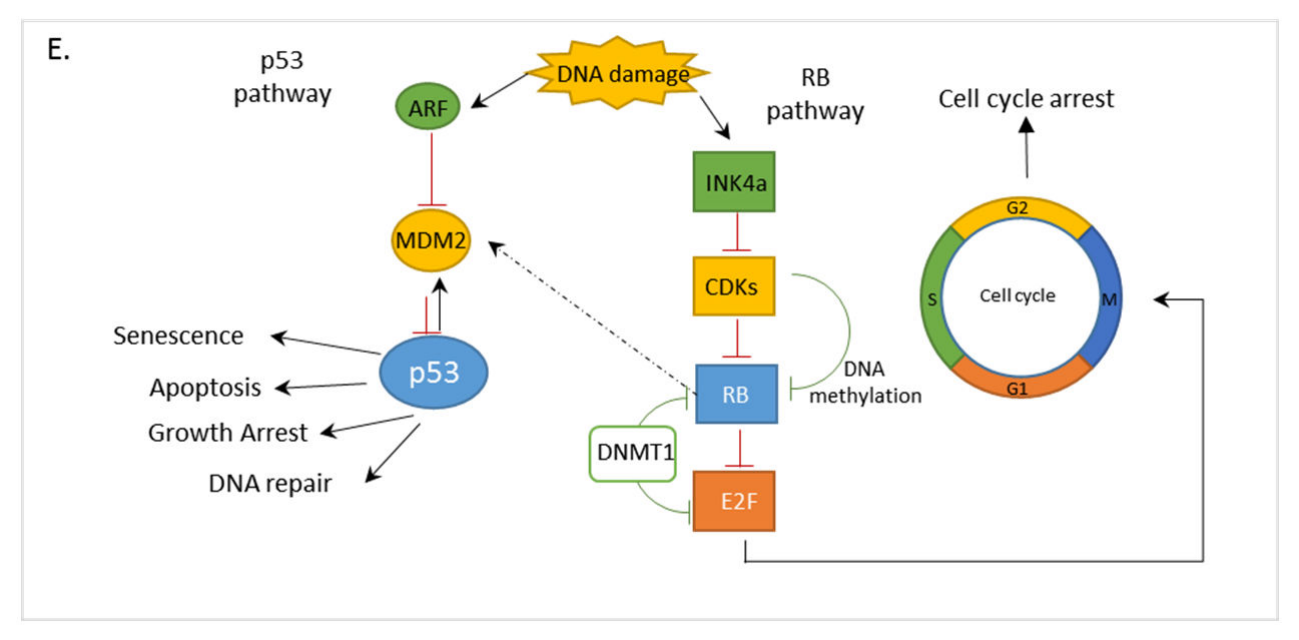
Hypermethylated genes in the dysregulation of the retinoblastoma (RB) tumor suppressor pathway in cancer. (**A**–**D**) Volcano plots showing the hypermethylation of breast (TCGA-BRCA; n_tumor_ = 785, n_normal_ = 95), colorectal (n_tumor_ = 313, n_normal_ = 38), lung (n_tumor_ = 370, n_normal_ = 42), and prostate cancers (n_tumor_ = 502, n_normal_ = 50) in the RB tumor suppressor pathway. The x-axis is log_2_ fold change of DNA methylation (beta values), whereas the y-axis shows the −log_10_ of the *p* values for each CpG site representing the strength of the association. Each point represents a CpG site. Dashed lines indicated cutoffs for significance, where *p* < 0.05 and log_2_ (Fold Change) > 1 (hypermethylation) or log_2_ (Fold Change) < −1 (hypomethylation). The number of hypermethylated CpGs in each cancer type is shown in green, and hypomethylated CpG sites are shown in red. (**E**) Schematic of the RB tumor suppressor pathway and its interaction with the p53 pathway. The p53 and RB pathway are two main tumor suppressor pathways that control cellular response to DNA damage and oncogenic stimuli. Each pathway consists of several regulators and effectors. For simplicity, only four main components of each pathway are presented. In the RB pathway, stress signals such as DNA damage and oncogenes induce the upregulation of INK4a. Ink4a inhibits cyclin-dependent kinase (CDK)s, which in turn inactivates RB via phosphorylation or DNA hypermethylation. This inhibits E2F activity by forming the complex RB-DMNT1-E2F, which acts on the G1 and S phase of the cell cycle, leading to cell cycle arrest. RB also regulates p53 activity through a trimeric p53-MDM2-RB complex. In this pathway, DNA hypermethylation occurs at the RB gene, leading to transcriptional repression of the RB gene and disruption of the M phase of the cell cycle leading to oncogenesis. Alternatively, the epigenetic silencing of RB may also negatively influence the function of the MDM2 protein in the p53 pathway, leading to inhibition of key cellular functions (e.g., apoptosis, DNA repair).

The retinoblastoma (RB) pathway plays an important role in the cell cycle progression by acting as a regulator of cell proliferation and growth suppression. To identify differential methylation of genes associated with the RB suppressor pathway, DNA methylation data (i.e., β values) from primary tumor and corresponding normal tissue samples were extracted for comparative analysis. This analysis focused on the CpGs within the promoter regions (i.e., CpG islands) of the four analyzed cancers (Figure 4), and identified the following number of hypermethylated locis (FDR < 0.05, fold change > 1): TCGA-BRCA (n_hippo_ = 318, n_wnt_ = 343), TCGA-COAD (n_hippo_ = 349, n_wnt_ = 426), TCGA-LUSC (n_hippo_ = 219, n_wnt_ = 244), and TCGA-PRAD (n_hippo_ = 218, n_wnt_ = 223).

### 3.2. Identification of CpG Sites Using the MethylMine Pipeline

**Figure 5:**
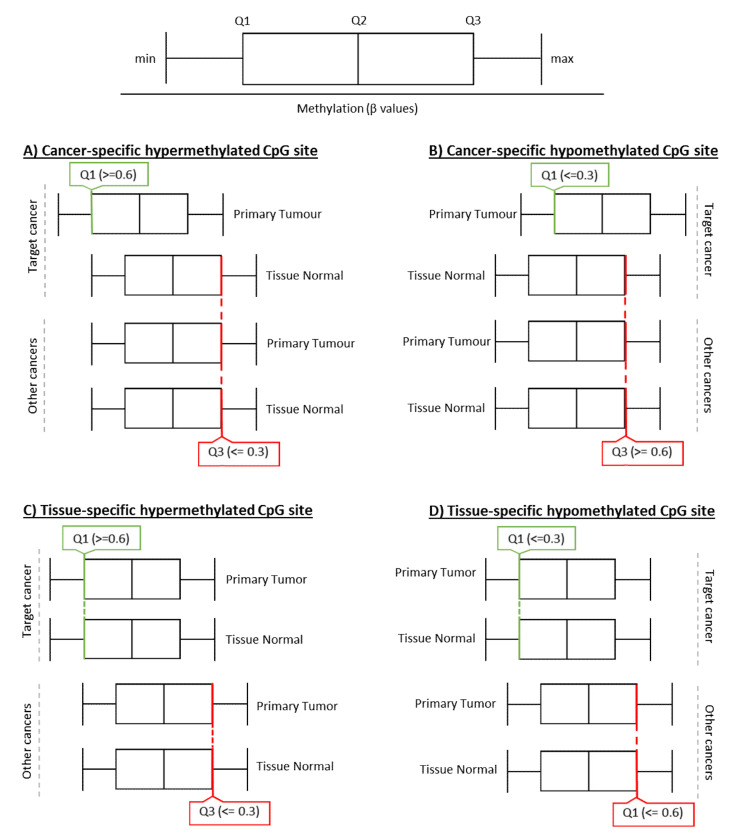
Overview of the Quartile Parsing methodology in the MethylMine framework. (**A**) Cancer-specific hypermethylated CpG site: more than 75% (Q1) of the primary tumor CpG beta values are above 0.6 (60% methylated), while more than 75% (Q3) of the normal tissue (target cancer), and the primary tumor and normal tissue of other cancers, are less than 0.3 (30% methylated). (**B**) Cancer-specific hypomethylated CpG site: more than 75% of the primary tumor samples of the target cancer are less than 0.3 (30% methylated), while more than 75% of normal tissue of the target cancer, and primary tumor and normal tissue of other cancers, are more than 0.6 (60% methylated). (**C**) Tissue-specific hypermethylated CpG site: more than 75% (Q3) of the normal tissue and primary tumor of the target cancer are more than 0.6 (60% methylated), (**D**) Tissue-specific hypomethylated CpG site: more than 75% (Q3) of the normal tissue and primary tumor of the target cancer is less than 0.3, and more than 75% of other cancers, are more than 0.6 (60% methylated).

Given that comparative analysis identified hypermethylated genes that were commonly found in breast, colorectal, lung and prostate cancer samples from the TCGA initiative, we sought to propose a method that could identify pathway-associated genes with a distinct methylation profile between the tumor and matching normal tissue. The MethylMine pipeline was written in the Python (3.8+) language to identify distinct methylation biomarkers based on a feature selection method. This pipeline filters for differentially methylated CpG biomarkers by comparing the Q1 (25th percentile) and Q3 (75th percentile) of sample methylation of tumor and normal tissue samples, respectively, across each CpG site (outline of MethylMine outlined in Figure 5). To identify epigenetic biomarkers, which are differentially methylated between breast (TCGA-BRCA), colorectal (TCGA-COAD), lung (TCGA-LUSC), and prostate (TCGA-PRAD) cancers or tissues, the MethylMine pipeline was used to compare the methylation values of both (primary) tumor and normal tissue samples (sourced from the same patient) across each CpG site, and the total number of predicted biomarkers was summarized in Table 3.

For cancer-specific hypermethylated CpG sites, the Q1 of the primary tumor (of target cancer) are above 0.6 (60% methylated), while Q3 of the normal tissue (of target cancer) and the Q3 of the primary tumor and normal tissue of other cancers are less than 0.3 (30% methylated). For cancer-specific hypomethylated CpG sites, the Q3 of the primary tumor (of target cancer) are below 0.3 (30% methylated), while the Q1 of the normal tissue (of target cancer) and the Q1 of the primary tumor and normal tissue of other cancers are more than 0.6 (60% methylated). For tissue-specific hypermethylation, the Q1 of the primary tumor and normal tissue of the target cancer is more than 0.6 (60% methylated), and the Q3 of the primary tumor and normal tissue of the target cancer is less than 0.3 (30% methylated). For tissue-specific hypomethylation, the Q3 of primary tumor and normal tissue of the target cancer is less than 0.3 (30% methylated), and the Q1 of the primary tumor and normal tissue of other cancers are more than 0.6 (60% methylated). These criteria were selected based on previously analyzed data (not shown), and has been found to be effective in selecting for CpG locis with distinct methylation profiles between the tumor and matching tissues and can be adjusted to suit the user’s needs. Using this algorithm, a list of 1053 CpG sites were found to be differentially methylated between the four cancers. A list of the MethylMine extracted probes is summarized in Table S1. Interestingly, the MethylMine pipeline returned a limited number of CpG loci that underwent cancer-specific hypermethylation in breast and lung cancer. Furthermore, there appeared to be a limitation in the number of methylation biomarkers that underwent tissue-specific hypermethylation. This was in contrast to previous studies that reported hypermethylation of a number of promoter-associated CpGs in both tumor tissues and blood samples [72,73,74]. Given that the overall goal of the MethylMine pipeline was to screen for biomarkers with distinct methylation patterns between different tissue types (i.e., tumor and normal), it was not surprising that the pipeline did not return the same CpG biomarkers as previously reported. Furthermore, over 59% of CpGs are located in regions outside of the promoters of target genes. However, these non-promoter-associated CpGs (open sea CpGs) may still play a role in oncogenic regulated genes, as previous studies have suggested the methylation of CpGs within gene bodies has led to silencing of alternative promoters, retrotransposons, and other functional elements to maintain the efficiency of transcription [75,76,77].

### 3.3. Semi-Unsupervised Clustering Suggests MethylMine-Extracted Biomarkers Are Differentially Methylated

**Figure 6:**
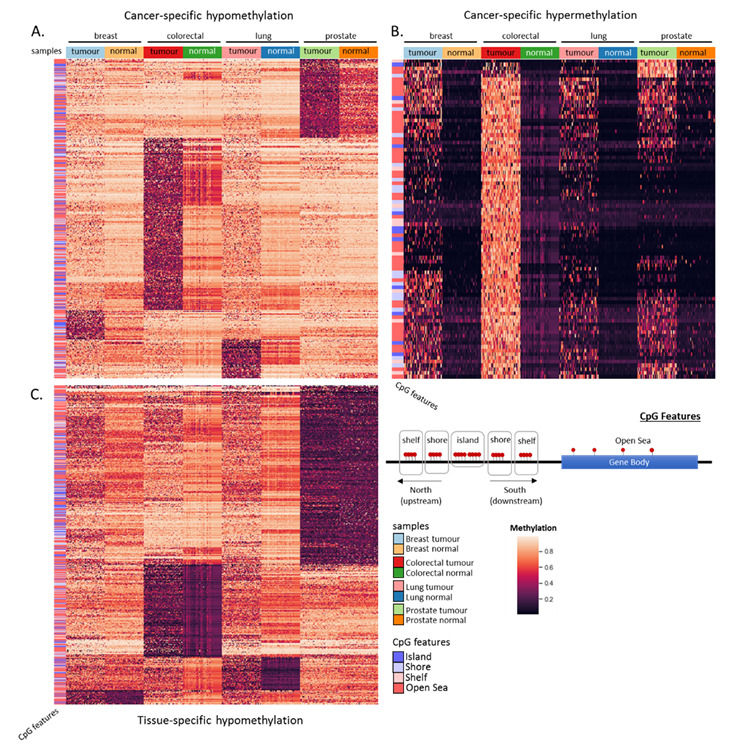
Visualization of MethylMine-filtered biomarkers of four cancers from the TCGA. Validation of MethylMine-filtered CpG sites across four cancers using semi-unsupervised clustering was performed with the following parameters applied on the CpG sites (row): method = average, metric = Euclidean. Filtered samples were further separated into (**A**) cancer-specific hypomethylation (samples from tumors of specific cancers are hypomethylated), (**B**) cancer-specific hypermethylation (samples from tumors of specific cancers are hypermethylated), (**C**) Tissue-specific hypomethylation (samples from tissues of specific sites are hypomethylated). CpG sites corresponding to different features are in the rows, while samples from either tumor or normal tissue of the four analyzed cancers are in the columns.

To determine if the MethylMine pipeline can be used to filter for differentially methylated CpG sites that were either cancer-specific or tissue-specific, the beta values of all filtered CpG sites of the primary tumor and normal tissue samples across the four cancers (breast, colorectal, lung and prostate) were extracted using a custom Python script (available through our github page). A semi-unsupervised clustering (method = average, metric = Euclidean) was performed separately on samples that displayed cancer-specific hypomethylation, cancer-specific hypermethylation, and tissue-specific hypomethylation (Figure 6), and related information summarized in Tables S2–S5. Given only two tissue-specific hypermethylation were detected in the using the MethylMine, pipeline (cg16171838 (ACVR1C), cg18061904 (ACVR1C)), they were excluded from the clustering analysis (Table S5).

Clustering of MethylMine-filtered CpG sites based on the feature selection (i.e., differences in quartiles) were defined by pronounced differences in the methylation of specific cancers and/or tissues, suggesting the pipeline used is effective in parsing for biomarkers with distinct methylation patterns between the different tissues and/or cancers. By further separating the filtered CpG samples into the MethylMine features (e.g., cancer-specific hypomethylation, tissue-specific hypermethylation), we were able to get a clearer picture of the CpG loci that were either hypo- or hypermethylated across the different tissues, when a semi-supervised clustering analysis was performed. This analysis presented panels of biomarkers that were specific for each cancer and/or tissue. To determine the association of the differentially methylated genes and its effects on the regulation of associated signaling pathways, a multi-omic data integration approach was used (Section 3.4).

### 3.4. Integration and Selection of Relavent ‘Omics’ Data Using the DIABLO Framework

To identify highly correlated multi-omic (methylation and mRNA) cancer-discriminating signatures between breast, colorectal, lung and prostate cancers, the DIABLO (Data Integration Analysis for Biomarker discovery) framework from the mixOmics package in R was used to integrate and analyze the methylation signatures of MethylMine-filtered CpG sites [78]. To determine if the MethylMine-extracted CpG targets played a role in the dysregulation of different signaling pathways during oncogenesis, the extracted epigenetic biomarkers were integrated with corresponding transcripts using a supervised learning approach within the DIABLO framework. The DIABLO framework performs N-integration by identifying multi-omics signatures that discriminate between the four cancers in a supervised analysis. To reduce the computational overhead of the program, only transcripts that were related to the filtered CpGs were included in the integrative analysis. Integration was performed using the sparse Partial Least Squares Discriminant Analysis (sPLS-DA), which enables the selection of the most predictive or discriminative features in the data that assist in the classification of samples. An illustration of the N-integrative supervised analysis using DIABLO of the four cancers was summarized in Figure S1. The N-integration analysis integrates different types of omics data measured on matched samples (i.e., mRNA and CpG extracted from the same specimen), and combined with the sPLS-DA, identified highly correlated sites for further enrichment analysis in order to identify dysregulated signaling pathways that may be mediated by epigenetic modifiers.

When both the RNA-Seq and CpG (meth) are plotted individually (Figure S1A), with the exception of the lung (TCGA-LUSC) (primary) tumor samples, there was a general overlap between the mRNA and CpG biomarkers. Indeed, when the differences between the mRNA and CpG (meth) were summarized in an arrow plot (Figure S1B), the long arrows indicated a weak agreement between the matching lung (TCGA-LUSC) (primary) tumor samples (i.e., mRNA and CpG). Similarly, a diagnostic scatterplot (plotDiablo) was used to determine the pairwise correlation between two omics datasets (i.e., CpG (meth) and mRNA) and has been modelled according to its design.

Here, we can see the MethylMine-filtered CpG sites are highly correlated with the integrated RNA-Seq mRNA transcripts (Figure S1C), with a correlation score of 0.96 (Pearson’s), which indicates a high correlation between the two. In this context, the score refers to the strong correlation between the location of the CpGs and the mRNA site and not the correlation between the level of DNA methylation and expression level of corresponding biomolecules. Additionally, the scatterplot suggests that the prostate cancer (TCGA-PRAD) tissues (both (primary) tumor and (tissue) normal) were discriminated from the other three cancers (i.e., breast, colorectal and lung). This observation was not surprising given MethylMine screened for biomarkers that had a pronounced difference in methylation between input cancers. Furthermore, a performance plot (Figure S1D) was used to evaluate and tune the performance of the sPLS-DA model for a large number of components (ncomp = 10) using repeat k-fold (k-fold = 3) cross validation. This plot displays the classification error rate when the number of samples in each group is unbalanced, and shows three predictive distances (max distance (dist), centroids dist, mahalanobis dist) as well as the overall error rate and the balanced error rate across the ten components. Although the plot suggested best performance can be achieved when ncomp = 3 (in both the overall and balanced error rate), only data from the first two components underwent further analysis given this appeared to provide a lower error rate (data not shown).

Following the performance analysis, the correlation between the CpG sites and RNA-Seq transcripts (mRNA) were visualized using two different methods. Firstly, a circos plot (Figure S1E) was used to visualize the correlation (positive or negative) between the level of methylation and RNA expression at each matched CpG-mRNA site, when the ncomp = 1–2 and the correlation cutoff = 0.6. With the exception of six matched CpG-mRNA sites that were shown to be positively correlated, all other matched sites were negatively correlated, and reflected the large number of cancer-specific hypomethulated sites presented by the MethylMine pipeline. Again, given the stringent criteria used to filter for differential methylation, the lack of cancer-specific hypermethylated sites detected was not unexpected. Interestingly, a previous study has found positive correlation between methylation and gene expression (i.e., association of hypermethylated CpG sites with expressed genes) of a number of sites using matching patient data from the TCGA [79], an observation that was reflected in the four positively correlated CpG-mRNA matches in Figure S1E, and closer inspection suggested these CpG sites are not promoter-associated, which indicates not all genes are affected by methylation in the same way, and the location of hypermethylation plays an important role in the final repression of the genes.

Moreover, a correlation circle plot (CCP) was used to highlight the correlation between the integrated mRNA and CpG sites. Corresponding to the circos plot, the CCP suggested there was a negative correlation between most CpG sites and the mRNA transcripts. The single CpG site (cg21393713), which overlapped with other mRNA sites, is located within the promoter region of the autism susceptibility gene (AUTS) 2 gene, a component of a polycomb group multiprotein complex required to maintain the transcriptionally repressed state of many genes, including the Hox genes throughout development. Although predominantly associated with neurological disorders (e.g., autism), dysregulation of AUTS2 has been implicated in both lung adenocarcinoma and prostate cancer [80].

Furthermore, a clustered image map (cim) was employed to visualize the multi-omic molecular signature expression for each sample (Figure S1G), with the level of correlation of expression shown by the color key. The cim showed a tight clustering of each sample type and different blocks of mRNA and CpG (meth) that are negatively correlated, which suggests they may play a role in the regulation of transcription of corresponding genes in these tissues, which will be further investigated using a network enrichment analysis. Multi-omic signatures that are highly correlated between the omics datasets were imported into the Cytoscape software package for analysis and visualization of complex pathways and networks [69].

### 3.5. Identification of Enriched Pathways in the Four Common Cancers

**Figure 7:**
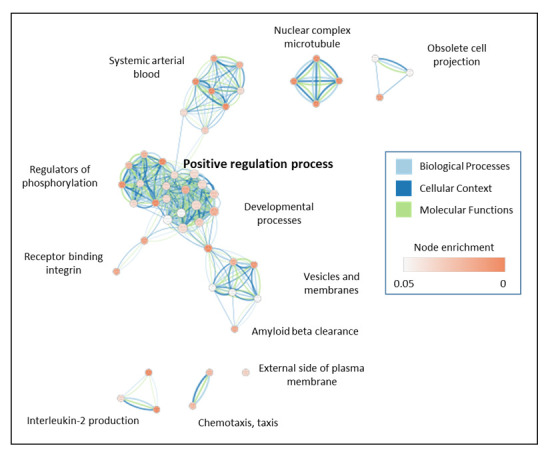
Representative biological and molecular networks derived from the TCGA dataset. Enrichment results were mapped as a network of gene sets (nodes) related by mutual overlap (edges), where the intensity in color indicates the level of enrichment of gene set. Node colour is proportional to the level of enrichment in each set and line color represents the pathways involved.

Pathway enrichment analysis has helped researchers gain a mechanistic insight into compiled gene lists by identifying biological pathways that are enriched in gene lists more than would be expected by chance [81]. It is an automated and statistically rigorous technique to analyze and interpret large gene lists from prior knowledge [82]. To investigate the function of the differentially expressed genes identified by the DIABLO framework in Section 2.3, a pathway enrichment map was constructed using the methodology outlined by Reinmand et al., 2019 [81]. In brief, the N-integrated CpG sites and mRNA targets (from DIABLO) were screened for enriched pathways by using the EnrichmentMap plugin of the cytoscape software, and an enrichment map (Figure 7) was generated, which grouped proteins with similar pathways together. Associated genes associated with each pathway were summarized in Table S6.

The enrichment map showed pathways that were dysregulated as a result of abnormal methylation between the four cancers screened—breast, colorectal, lung and prostate. The enrichment analysis appeared to highlight processes that are essential in normal cellular processes such as regulators of phosphorylation (most widespread type of post-translational modification used in signal transduction) and receptor-binding integrin (regulates cell adhesion, migration, invasion, and cell survival). We found that most of the genes were connected together to form a giant linked network component involved in positive regulatory processes. Interestingly, the enrichment analysis (Figure 7) did not highlight any of the commonly disrupted pathways (e.g., ARF-MDM2-p53, NRF2-KEAP1 and PI3K pathways) found in most cancers, despite our initial comparative analysis, which suggested the hypermethylation of associated genes across the four canonical pathways in this manuscript (Section 3.1). Therefore, we sought to determine if any of the aberrantly methylated genes detected using the current pipeline corresponded to a specific oncogenic signaling pathway and could therefore be used as potential epigenetic biomodifiers.

### 3.6. MethylMine-Filtered CpG Sites Are Involved in the Disruption of the KEAP1-NRF2 and PIK3 Signalling Pathway in Cancer

**Figure 8:**
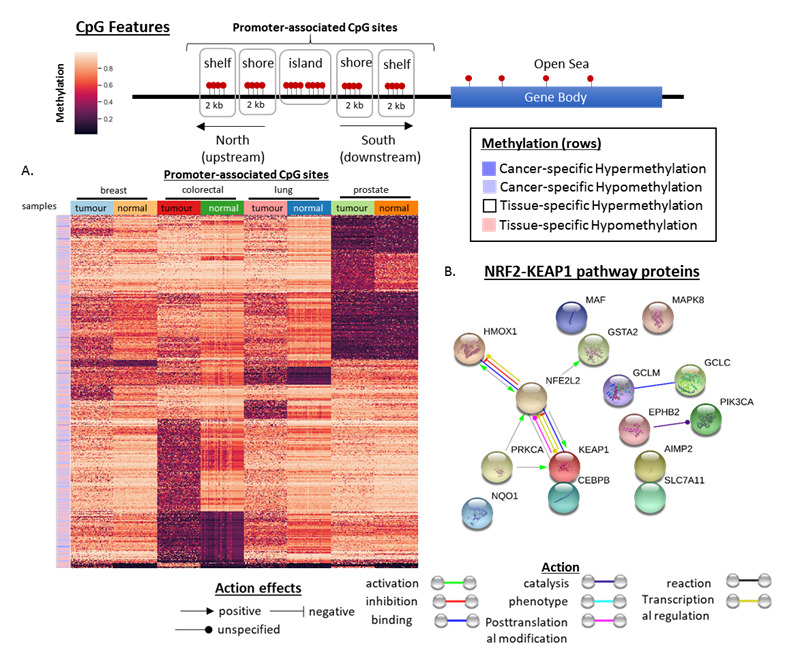
Visualization of MethylMine-filtered methylation biomarkers that overlap with the NRF2-KEAP1 and PIK3 pathway. (**A**) Visualization of differentially methylated CpG sites within the promoter associated CpG sites. CpG sites corresponding to different types of methylation are in the rows, while samples from either tumor or normal tissue of the four analyzed cancers are in the columns. (**B**) Protein-protein network analysis of filtered proteins from the MethylMine pipeline-filtered probes that found 15 corresponding genes that are associated with the NRF2-KEAP1 and PIK3 pathway. The mode of action between each protein (node) is described by the different colors of the lines, while the effects of the action are summarized by the direction of the arrow or symbol.

To determine if MethylMine identified differentially methylated CpG sites (in the four cancers) that are dysregulated in a common oncogenic signaling pathway, samples of filtered CpGs were analysed using a semi-supervised analysis. To reduce noise, only CpG sites located within the promoter region of associated genes were analyzed (Figure 8A). Clustering using this methodology suggested that the MethylMine pipeline could be used to filter for biomarkers that showed distinct methylation patterns between the cancers and different tissues. Furthermore, to determine if the differentially methylated proteins overlapped with any genes within the canonical pathways surveyed in Section 3.1, the STRINGS protein-protein interaction analysis [67] was performed to determine the functional interactions and identify the most related pathway(s). Interestingly, only 15 proteins, which intersected with the NRF2-KEAP1 and PIK3 signaling pathway, were identified (Figure 8B).

Combined with the pathway analysis, the heatmap revealed the differential methylation of different tumors, such as CYBA in prostate cancer and CSMD2 in lung cancer, which were previously reported to be involved in the progression of the respective cancers by being involved in the production of superoxide and/or hydrogen peroxides [83], which plays a role in the NRF2-KEAP1 pathway.

## 4. Discussion

Aberrant DNA methylation is a widespread feature in cancer that can lead to the transcriptional regression of genes and alterations in different biological and molecular pathways that may play a role in the progression of oncogenesis. Although many studies have described the effects of aberrant DNA methylation on the onset and progression of cancer [84], a survey of literature suggests there is still a limitation in studies that links abnormal methylation to the alterations of different signaling pathways that commonly occur in cancers across different anatomical sites [85,86]. In this study, we investigated the methylation profile of four canonical oncogenic signaling pathways by revisiting the TCGA dataset of the four most common cancers in the world [61], and explored the pathway-associated genes that may be epigenetically-driven among cancers across the different cancers, and could therefore be used as potential drug targets to correct the altered signaling pathways and the possibility of combination therapy to target multiple sites in patient care. By filtering for CpG sites that corresponded to genes associated with each of the oncogenic signaling pathways described in this manuscript, we identified cohorts of CpG sites that are universally hypermethylated in the tumor samples of the four TCGA cancers screened and could serve as potential pan-cancer biomarkers.

The ARF-MDM2-p53 pathway (commonly referred to as the p53 pathway) has become the focus of many investigations due to its importance in tumor suppression, as shown by its genetic inactivation in more than half of human cancers and its functional impairment occurring in most of the remaining cancers, suggesting the reactivation of p53 as a treatment option that is potentially effective for a wide range of human cancers [34,87,88,89]. This process is involved in protecting cells against genetic instability by initiating cell cycle arrest, apoptosis, or senescence in response to cellular stress and DNA damage [90] (Figure 1E), and creates effector functions that impact on most of the hallmarks of cancer as described by Hanahan and Weinberg [1]. Previous studies have suggested that the inactivation of ARF (p14) through epigenetic silencing plays an important deregulating mechanism of the ARF-MDM2-p53 pathways, leading to a restriction of the tumor-suppressing activity of p53 [91]. However, studies that focused on the effects of DNA methylation in the disruption of this pathway are still limited. Therefore, we compared the DNA methylation profile in the primary tumor and normal tissue samples of the top four cancers (breast, colorectal, lung and prostate) in order to identify ARF-MDM2-p53 pathway-associated genes, which may be universally hypermethylated in the cancers investigated. Volcano plots was used to screen for differential methylation patterns in the four TCGA cancers (Figure 1A–D). This analysis suggested the hypermethylation of TWIST1, CDKN2A (ARF2), PIK3R5 and TBX2 are present in cancers of the breast, colon, lung and prostate. Hypermethylation of the associated proteins (e.g., TWIST1) individually have multiple biological functions in various cancers [70,71] by acting on different components within the p53 pathway (Figure 1E), and could therefore serve as potential targets for cancer therapeutics.

The KEAP1-NRF2 and PI3K pathway is the major regulator of cytoprotective responses to endogenous and exogenous stresses caused by reactive oxygen species (ROS) and electrophiles [92]. ROS defines any oxygen-containing radicals that are capable of independent existence with one or more unpaired electrons and act as mediators of both physiological and pathophysiological signal transduction [93]. Elevated levels of ROS are a common hallmark of cancer progression and have been detected in cancer cells due to an increase in metabolic activity, cellular signaling, activation of oncogenes, and increased enzymatic activity of oxidases (including cyclooxygenases, lipoxygenases, and thymidine phosphorylases). The NRF2 protein is a master regulator of redox homeostasis and plays a key role in regulating a wide array of genes for antioxidant and detoxification enzymes [94]. A large number of existing literatures have recognized the extensive alterations in this signaling pathway and its potential as drug-able cancer targets [95,96]. Activation of the PI3K pathway (induced by ROS) leads to the accumulation of the transcription factor NRF2 that has a protective role in metabolic pathways [97]. In many cancers, the NRF2 and KEAP1 will undergo hypermethylation, leading to a repression of expression of the KEAP1 protein and the release of NRF2 [98,99,100]. This epigenetic modification has been shown to regulate the expression of cervical cancer, renal cell carcinoma and glioblastoma [100,101]. There is a clear implication of DNA methylation on the complex regulation of the KEAP1/NRF2 couplet (most frequent mechanism of KEAP1 silencing in solid tumors) required in the NRF-PI3K pathway. Furthermore, epigenetic reprograming of NRF2 can lead to its reactivation and subsequent induction of downstream target genes involved in cellular protection (Figure 2E), further suggesting the use of KEAP1 within the NRF2 pathway as a potential target for cancer treatment. Comparative analysis of methylation sites associated with genes within the KEAP-NRF2 and PI3K pathway in this study suggested the MAF transcription factor and NFE2L2 (NRF2) are hypermethylated in the four TCGA cancers screened. As MAF forms a couplet with NRF2 and binds to the antioxidant response element (ARE) in the regulatory regions of target genes to promote the transcription of cytoprotective genes, the methylation of MAF and NRF2 could serve as potential therapeutic targets to treat the analyzed cancers in this study.

The Hippo and Wnt signaling pathways play a crucial role in maintaining tissue homeostasis and organ size by orchestrating cell proliferation, differentiation, and apoptosis. These pathways are highly complex and have been frequently found to be dysregulated in human cancers. The Hippo pathway consists of a large network of proteins that controls the growth of different tissue during development and growth, as well as in different diseases such as cancer [102], while the Wnt (wingless-related integration site) signaling pathway orchestrates various biological processes, such as cell proliferation, differentiation, organogenesis, tissue regeneration, and tumorigenesis [103,104,105,106,107]. The importance of the Hippo and Wnt pathway in human cancers was first demonstrated when the human tumor suppressor adenomatous polyposis coli (APC) protein was found in association with β-catenin. In these studies, it was found that the APC protein is mutated and inactivated in colorectal cancers, typically in the early stages of cancer development [108]. Methylation analysis of genes associated with either the Hippo or Wnt pathway revealed hypermethylation of a wide range of genes that are shared between the two pathways across the four cancers screened (Figure 3A–D). This cross-talk between the Hippo and Wnt signaling pathways has been explored in a number of studies, and the hypermethylation of these common genes between the two pathways may provide novel therapeutic targets for cancer treatment.

The RB pathway has been a focus in cancer therapy [109,110], as several components of this pathway, including p16Ink4a, cyclin D1 and RB, are frequently deleted or mutated during the progression of cancer. The hypermethylation of CpG 106, which overlaps the promoter and exon 1 of RB, was observed to be methylated in most retinoblastoma tumors, thereby suggesting the implications of hypermethylation of promoter-associated CpGs of tumor suppressors in tumorigenesis [111]. Subsequently, hypermethylation of CpG sites at the 5′ end of RB1 promoter were found to lead to reduced gene expression [112,113]. Since then, many studies have recorded the role of RB1 promoter hypermethylation in different cancers [114,115,116,117,118], most commonly in gliomas (the most common cancer of the brain) [119]. Specifically, glioma-CpG island methylator phenotype tumors are associated with hypermethylated promoters and IDH 1 mutations [120]. A recent study by Daniel et al. revealed a connection between the methylation at the MGMT (methylguanine DNA methyltransferase) promoter and a hypermutator phenotype secondary to mismatch repair deficiency occurring in glioma. Therefore, the DNA methylation of profile of the RB tumor suppressor pathway of the four common cancers was visualized using a volcano plot with hypermethylated CpGs of genes within thin associated protein highlighted in green on the left of each related plot (Figure 4A–D). Hypermethylated genes were observed across the four cancers, such as ZNF655, RB1, and TFDP2, which have previously been reported to be epigenetic diagnostic biomarkers for various cancers (including breast and colorectal cancers) [121,122,123].

Finally, to identify genes that are differentially methylated between the four cancers screened, we used a combination of our differential methylation parsing pipeline—MethylMine—and unsupervised clustering to identify an initial panel of methylation sites, which are differentially methylated across the four selected cancers—breast, colorectal, lung and prostate. Furthermore, multi-omic integration and pathway enrichment analysis were conducted to identify signaling pathways, which may be dysregulated during the onset of the four cases as a result of these aberrant methylations. In contrast to previous studies that used inferential statistical methods (e.g., t-test, ANOVA, etc.) to mine for differentially methylated genes between different sample groups, the MethylMine pipeline extracted differentially methylated biomarkers by comparing the quartiles of methylation values at each probe across both (primary) tumor and normal tissue samples of selected cancers. The assumption here is that most of the methylation pattern of at least 75% of the samples across a CpG site would be above a certain beta value (i.e., 0.6) if the CpG was hypermethylated. As opposed to inferential statistical methods, we found biomarkers extracted using the MethylMine pipeline returned biomarker panels that presented a marked difference between the different tissues and cancers (Figure 2), and required less post hoc adjustments to reach the final results. As methylation of CpGs within gene bodies has been demonstrated to lead to the silencing of alternative promoters [75,76,77], these epigenetic targets could serve as therapeutic targets to correct the function of downstream pathways.

Interestingly, given the same dataset, the MethylMine pipeline extracted different a set of biomarkers then the comparative analysis performed above (Figure 1, Figure 2, Figure 3 and Figure 4), as this feature-based method screened for biomarkers that showed a distinct methylation profile between different cancers and/or tissues (Figure 6). Therefore, it was not surprising the above pipeline extracted a set of different methylation biomarkers to the hypermethylated CpG sites identified during the comparative methylation screening where the main selection criteria was the fold change in methylation between the tumor and normal tissue samples. Furthermore, given the stringent criteria used to filter for CpGs sites with distinct methylation patterns (Figure 5), it was not surprising MethylMine did not detect the same CpG biomarkers (with a lower difference in methylation) as previously reported [72,73,74] or in the comparative analysis.

To determine if MethylMine-extracted CpG sites influenced the activity of associated genes, the 1053 CpG extracted CpG sites were integrated with the corresponding RNA-Seq transcriptomic data using the N-integration method within the DIABLO module of the mixOmics package. This integration allowed the merge of CpG sites with its corresponding mRNA transcripts so we could determine if the expressed genes (mRNA transcript) were influenced by the methylation pattern of corresponding CpG loci. The integrated data was then analyzed using an enrichment analysis to identify differentially methylated pathways that were disrupted in the four cancers. Interestingly, the enrichment analysis highlighted pathways that were predominantly involved in the regulation of developmental and phosphorylation processes (Figure 7). This suggested that the developmental and regulatory processes, which are important in the homeostatic state of healthy cells, are disturbed during oncogenesis. This builds up the complexity of cancer signaling pathways, therefore complicating the process of finding treatments to target different cancers.

Given this, we sought to determine if MethylMine identified differentially methylated biomarkers that overlapped with any of the four signaling pathways analyzed. By using a combination of interaction analysis and semi-supervised clustering, we identified a total of 15 differentially methylated genes, which corresponded to the NRF2-KEAP1 and PIK3 pathway (Figure 8B). Furthermore, differentially methylated genes, which play a role in the production of reactive oxygen species (ROS), were identified in tumors of the breast, colon and prostate, respectively. Reactive oxygen species and hypoxia have been viewed as major hallmarks of cancer due to their ability to influence the tumor micro-environment and initiate cancer angiogenesis and metastasis [1]. The NRF2-KEAP1 and PIK3 pathway are involved in the regulation of expression of cytoprotective genes under stress conditions (Figure 2E), such as presentation of the ROS and hypoxia during disease [92]. This finding supports the ability of MethylMine to identify differentially methylated CpG sites corresponding to genes that are associated with canonical signaling pathways in various cancers.

## 5. Conclusions

Despite the boost in the post-genomic era, there is an urgent need to explore the large volume of methylation data that is curated in publicly available databases in order to explore the commonalities and differences between signaling pathways that may drive the progression of cancer. To explore the use of epigenetic modifiers in various cancers, we revisited the TCGA dataset, and performed a comparative methylation analysis, which suggested the hypermethylation of genes associated with four canonical oncogenic signaling pathways. Although they have individual functions, all four pathways shared common proteins and transcription factors, which could be targeted by different biomodifiers to diagnose or treat cancer. As changes in DNA methylation may result in altered signaling pathways leading to the progression of cancer, here we demonstrated the potential of altering methylation-based targets within these pathways to change their phenotypic expressions, and ultimately switch the pathway back into a non-disease state. Furthermore, we described a differential methylation parsing pipeline MethylMine that filters for differentially methylated biomarkers by comparing the different quartiles between the (primary) tumor and normal tissue of each CpG site of corresponding cancers. By integrating the corresponding RNA-Seq transcript data, we were able to screen for transcripts and by extension, genes that were expressed and enriched in different signaling pathways. As the MethylMine framework filtered for differentially methylated CpGs between different cancer types, we propose this algorithm can be used to screen for cancer-specific CpG markers. Our analysis suggested the pipeline used is effective in filtering for differential methylation of genes associated with the NRF2-KEAP1 and PIK3 pathway, which acts as a major regulator of cytoprotective responses to endogenous and exogenous stresses via ROS. Additionally, genes that encode for ROS-generating NADPH oxidases (e.g., NOX, CYBA) were also detected using the current pipeline and have been found to play a role in most cancers. Therefore, the MethylMine pipeline is able to screen for differentially methylated biomarkers with distinct changes in methylation profile between the different cancers and tissues. Additionally, this algorithm will be improved in future versions to screen for pan-cancer biomarkers in order to mine for biomarkers that are shared between different cancers to uncover epigenetic modifiers that can be used to target multiple cancers. As such, the use of DNA methylation as an epigenetic modifier is promising, opening an avenue for identification of novel drug targets, resistance patterns, and the development of combination therapies to treat multiple targets across various signaling pathways to target different cancers.

## Figures and Tables

**Table 1:** Summary of DNA methylation biomarkers in five common cancers that is approved for clinical use.

Cancer	Methylated Gene	Description	References
Breast	↑AKR1, ↑HOXB4, ↑RASGRF2, ↑RASSF1, ↑HIST1HC3, ↑TM6SF1, ↑GSTP1, ↑RARβ2,	Predictive of early stage metastatic breast cancer	[36,37,38,39]
Ovarian	↑RASSF1A, ↑RASSF2A, ↑ESR1	Predictive of early stage carcinogenesis	[40,41,42]
Colorectal	↑FBN2, ↑MAL, ↑SST, ↑SFRP2, ↑NDRG4, ↑VIM	Predictive of malignant colorectal cancer in tissue and blood	[43,44,45,46,47]
Lung	↑p16, ↑DAPK, ↑PAX5b, ↑GATA5, ↑RASSF1A	Predictive of non-small cell lung carcinoma	[48]
Prostate	↑MCAM, ↑ERα, ↑ERβ, ↑PCDH10, ↑CDH13	Predictive of early stage prostate cancer	[49,50,51]

↑ hypermethylation.

**Table 2:** Overview of multi-omics datasets analyzed in this study.

Dataset	Sample Size in Each Tissue Type		Omics	Number of Variables
	Primary Tumor	Normal Tissue		
Breast Cancer (TCGA-BRCA)	780	95	CpG	485,000
			mRNA	60,482
Lung Cancer (TCGA-LUSC)	270	42	CpG	485,000
			mRNA	60,482
Colorectal Cancer (TCGA-COAD)	313	38	CpG	485,000
			mRNA	60,482
Prostate Cancer (TCGA-PRAD)	502	50	CpG	485,000
			mRNA	60,482

**Table 3:** Tally of differentially methylated CpG sites predicted by MethylMine.

Sites	Breast	Colorectal	Lung	Prostate
Cancer-specific hypermethylation	0	82	0	5
Cancer-specific hypomethylation	42	250	56	116
Tissue-specific hypermethylation	0	2	0	0
Tissue-specific hypomethylation	23	145	53	279

## Supplementary Materials

The following are available online at https://www.mdpi.com/2072-6694/12/11/3199/s1, Figure S1: Illustration of N-integration supervised analysis with DIABLO. Table S1: List of enriched pathways and genes found in the enrichment analysis; Table S2–S4: Tables of CpG sites that are differentially methylated between the four different cancers; Table S6: List of MethylMine-filtered probes. Supplementary methylation information: results of comparative methylation analysis of CpGs across different canonical oncogenic signaling pathways.
