# Supplementary material for: Regulation of Canonical Oncogenic Signaling Pathways in Cancer via DNA Methylation
Source: Cancers (Basel). 2020 Oct 30;12(11):3199. doi: 10.3390/cancers12113199 (PMC7692324; doi:10.3390/cancers12113199)
Supplement: Supplementary file 1 [file cancers-12-03199-s001.zip › cancers-933492_Supplementary_Files/cancers-933492_Supplementary_Figure_S1.pdf]

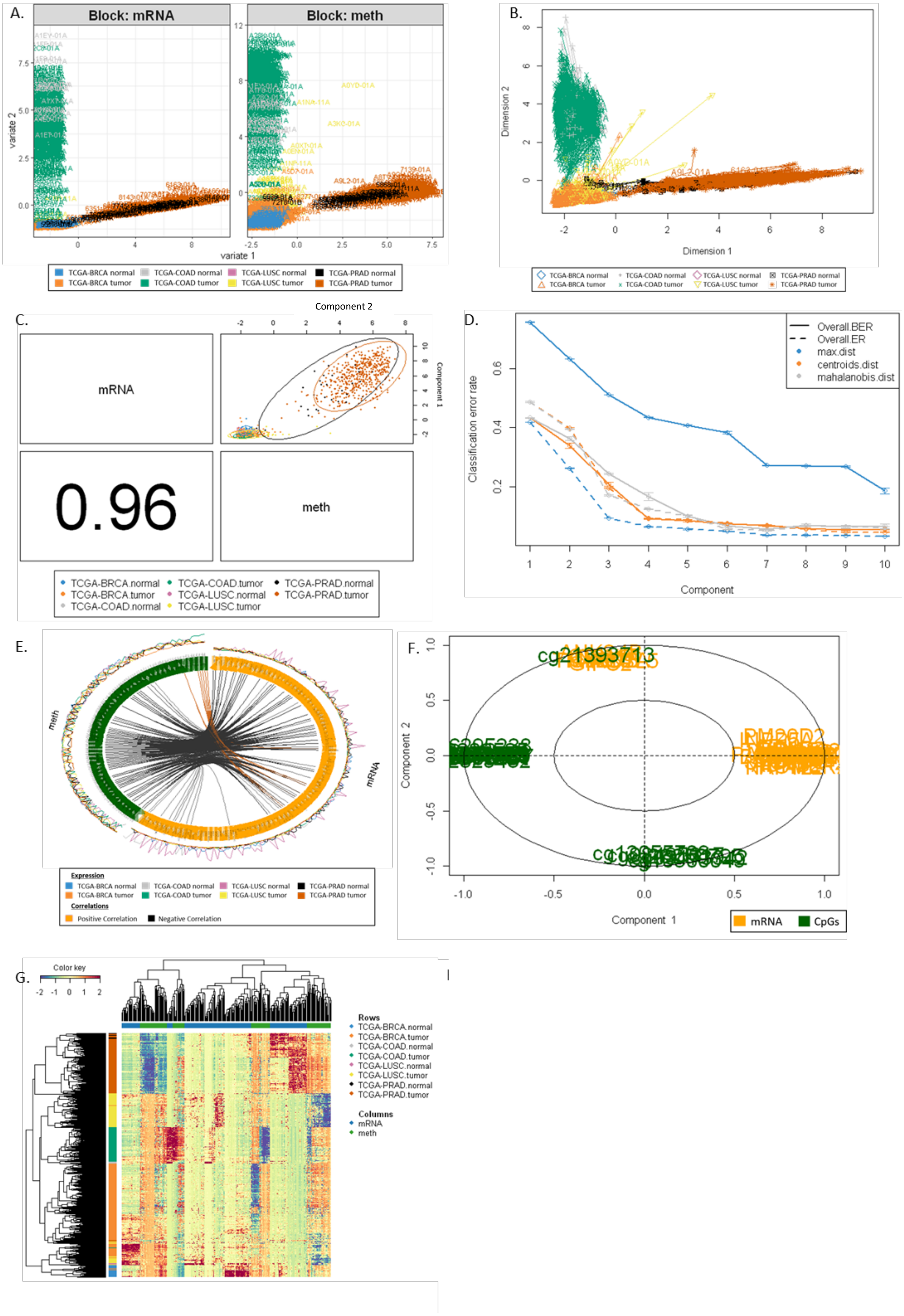

Supp Figure 1: Illustration of N-integration supervised analysis with DIABLO. **A**. sample plot per data set highlighting the integrated CpG and mRNA targets, **B**. Arrowplot of integrated data showing the relationship between samples summarized with a combination of selected CpG and transcripts pointing towards its outcome category, **C**. Diagnostic scatterplot displaying the first component of each dataset (mRNA and meth (CpG)), **D**. classification performance per component (overall and Balanced Error Rate (BER)) for three prediction distances using repeated stratified cross-validation (10 x 5 fold CV), **E**. Circos plot shows the positive and negative correlation (r > 0.6) between selected features as indicated by the orange (positive) and black (negative) links, feature names appear in the quadrants, with the level of correlation of each tissue type above each quadrants, **F**. Correlation Circle plot representing each type of selected features (CpG and mRNA), **G**. Clustered Image Map (Elucidian distance, Complete linkage) of the multi-omics signatures. Samples are represented in rows, selected features on the first component in columns,
